# Integrated Fruit Ripeness Assessment System Based on an Artificial Olfactory Sensor and Deep Learning

**DOI:** 10.3390/foods13050793

**Published:** 2024-03-04

**Authors:** Mingming Zhao, Zhiheng You, Huayun Chen, Xiao Wang, Yibin Ying, Yixian Wang

**Affiliations:** 1 School of Biosystems Engineering and Food Science, Zhejiang University, Hangzhou 310058, China; 2 Key Laboratory of Intelligent Equipment and Robotics for Agriculture of Zhejiang Province, Hangzhou 310058, China; 3 ZJU-Hangzhou Global Scientific and Technological Innovation Center, Hangzhou 311200, China

**Keywords:** volatile organic compounds, fruit ripeness detection, artificial olfactory sensor, colorimetric sensing combinatorics, deep convolutional neural networks

## Abstract

Artificial scent screening systems, inspired by the mammalian olfactory system, hold promise for fruit ripeness detection, but their commercialization is limited by low sensitivity or pattern recognition inaccuracy. This study presents a portable fruit ripeness prediction system based on colorimetric sensing combinatorics and deep convolutional neural networks (DCNN) to accurately identify fruit ripeness. Using the gas chromatography–mass spectrometry (GC-MS) method, the study discerned the distinctive gases emitted by mango, peach, and banana across various ripening stages. The colorimetric sensing combinatorics utilized 25 dyes sensitive to fruit volatile gases, generating a distinct scent fingerprint through cross-reactivity to diverse concentrations and varieties of gases. The unique scent fingerprints can be identified using DCNN. After capturing colorimetric sensor image data, the densely connected convolutional network (DenseNet) was employed, achieving an impressive accuracy rate of 97.39% on the validation set and 82.20% on the test set in assessing fruit ripeness. This fruit ripeness prediction system, coupled with a DCNN, successfully addresses the issues of complex pattern recognition and low identification accuracy. Overall, this innovative tool exhibits high accuracy, non-destructiveness, practical applicability, convenience, and low cost, making it worth considering and developing for fruit ripeness detection.

## 1. Introduction

Fruits, essential in diets, are rich in vitamins, dietary fibers, polyphenols, and essential minerals, and are widely recognized for their health benefits [1]. However, it is crucial to consider that the quality of fruit is dictated by the ripening process which may take place during long transportation periods [2]. Fruit ripening results in changes in the color, texture, aroma, and nutritional quality of fresh fruit. It is also accompanied by the release of other volatile organic compounds (VOCs), which comprise diverse classes of chemicals [3], including water, esters, alcohols, aldehydes, ketones, lactones, and terpenoids. This diverse range of compounds can usually act together to reinforce or interfere with the sensor signal. Therefore, in such cases, it is more convenient to develop global selectivity devices, i.e., non-selective sensors, which are capable of detecting the differences in the breathing patterns of each class of fruit [4]. The ripening of a unique fruit may affect the others and, by extension, impact the overall product quality to final consumers. Therefore, effective fruit ripening monitoring is an attractive approach to ensure standard quality control and predict shelf life.

Sensor devices, such as artificial olfactory systems, are becoming increasingly popular for the detection of environmental pollutants [5], explosive compounds [6], food safety [7], and disease [8] due to their wide detection range, cost-effectiveness, portability, and non-destructiveness. Consequently, these systems offer a new method for monitoring fruit quality as a replacement for human specialists who take advantage of visual features in the grading process. The various gases emitted by fruit can interact with the dyes present on the colorimetric sensor array, resulting in the formation of a distinctive fingerprint pattern [9] that can be analyzed using pattern recognition techniques to predict the degree of fruit ripeness based on the observed changes in color. The development of naked-eye-detected artificial olfactory systems in smart food packaging has garnered considerable attention due to their ability to provide on-packaging indicators of fruit maturity and ripeness to consumers. However, the commercialization of artificial olfactory systems is currently hindered by the challenge of accurately distinguishing complex patterns. While colorimetric sensors (or opto-noses) are highly cross-reactive, they produce nonlinear and multidimensional datasets [10,11,12]. In addition, variable lighting conditions can significantly impair pattern discernment in image-based recognition systems, thereby reducing identification accuracy. Traditional statistical techniques such as principal component analysis (PCA) [13,14,15], hierarchical clustering analysis (HCA) [16,17,18,19], and discriminant analysis (LDA) [20,21,22], are unable to meet the needs for the accurate extraction and analysis of complex image datasets.

To tackle these challenges, the deep convolutional neural network (DCNN) offers a powerful method compared with traditional statistical techniques. PCA and HCA primarily focus on descriptive analysis, limiting their predictive capabilities, whereas DCNNs are more flexible and tailored for classification, making them more suitable for these predictive tasks. Traditional classification methods like LDA are fundamentally linear and often fall short when applied to nonlinear data like images. In contrast, DCNNs excel by extracting complex features and learning intricate patterns, thereby effectively processing multidimensional image data and reducing environmental variability [23,24]. Guo et al. utilized ResNet101 for the colorimetric detection of meat freshness [25], achieving an impressive accuracy of 98.5%. They used 20 unique types of porous nanocomposites made of chitosan, dye, and cellulose acetate and integrated a DCNN into a smartphone application to rapidly identify food freshness. Huang et al. combined DCNN, PAN-NSS, and an app to develop a rapid, long-lasting nitrite sensor system in food products [26]. They extended sensor life from 7 to over 30 days and enhanced the accuracy of food classification to 91.33–100.00%. Ma et al. developed a colorimetric sensor array using six chromogenic indicators loaded with UiO-66-OH to assess chicken freshness [27]. They used the WISeR-50 algorithm and achieved a 98.95% accuracy rate. With the advancement of deep learning, the densely connected convolutional network (DenseNet) [28] has emerged as a novel algorithm with distinct advantages in complex image processing. The efficient use of dense connections not only enhances feature extraction and reuse but also minimizes overfitting. Thus, this study integrated DenseNet with colorimetric sensor arrays to evaluate fruit ripeness.

Therefore, the original objectives of the present work were to simulate the human olfactory system (Figure S1a), and establish a low-cost, non-destructive, high accuracy fruit ripeness prediction system, employing colorimetric sensor techniques integrated with DenseNet (Figure S1b). The GC−MS method was first used to determine the characteristic VOCs of mango, peach, and banana at different ripening stages. Then, colorimetric sensing combinatorics with multiple dyes were used to enable the sensitive detection of various characteristic VOCs and formed scent fingerprints. Finally, the DenseNet model was used to extract fingerprint information and categorize fruit ripeness.

## 2. Materials and Methods

**Figure 1:**
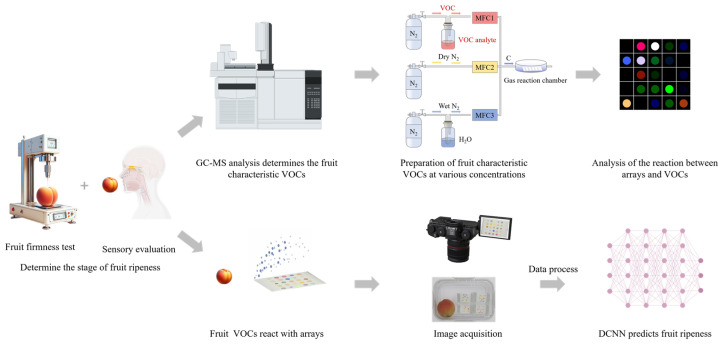
Flowchart of the methodological framework.

The overview of the methodological framework is presented in Figure 1. Initially, a fruit firmness test and sensory evaluation were employed to determine the stage of fruit ripeness. Subsequently, gas chromatography–mass spectrometry (GC-MS) analysis was utilized to determine the characteristic VOCs emitted by the fruits at different stages of ripening. Thereafter, the prepared characteristic VOCs with different concentrations were analyzed using the colorimetric sensor arrays and classified using the HCA method. The images of the colorimetric sensor arrays before and after their reactions with the VOCs released from fruits were captured using a camera (Canon EOS 70D, Canon Inc., Tokyo, Japan) and coupled with the DenseNet model to predict the fruit’s ripeness.

### 2.1. Chemicals and Materials

All the dyes (Table S1) were purchased from Sigma-Aldrich (Merck KGaA, St. Louis, MI, USA). Anhydrous ethanol (≥99.5%) and ethyl acetate were purchased from Sinopharm. Trans-2-hexenal and benzaldehyde were purchased from Sigma-Aldrich. Hexyl acetate, (+)-limonene, β-myrcene, 3-carene, and isoamyl acetate were purchased from Aladdin Biochemistry Technology Co., Ltd (Shanghai, China).

### 2.2. Fabrication of the Colorimetric Sensor Arrays

For the fabrication of the colorimetric sensor arrays, initially, 5 mg of each dye was dissolved in 5 mL of anhydrous ethanol using ultrasonication for 10 min to obtain a clear solution. Subsequently, a 3 cm × 3 cm Polyvinylidene fluoride (PVDF) membrane with pore diameters of 0.22 µm was used as a substrate. Then, a volume of 2.5 µL of each dye solution in 25 species were successively deposited onto the membrane’s surface in a 5 × 5 grid pattern, ensuring a 6 mm distance between adjacent dye spots. Following this, the prepared colorimetric arrays were heated at 75 °C on a heating plate for 30 min to guarantee the complete evaporation of ethanol, then cooled to room temperature for 30 min, and finally stored in a brown vacuum container filled with N_2_ for at least three days before measurements.

### 2.3. Fruit Firmness Test

A texture analyzer (TA−XT2i, Stable Micro Systems, Godalming, UK) equipped with a P/2 cylindrical probe was used to conduct puncture tests on the fruit with a testing speed of 1 mm s^–1^ and a penetration depth of 8 mm. Firmness was measured at three equidistant locations around the equator of the fruit. The mean firmness value of the fruit for each day was calculated from three replicates.

### 2.4. Sensory Evaluation of the Fruits

In this study, five experienced fruit farmers were invited to assess and score the ripeness of three fruits (mango, banana, and peach) using sensory analysis. Participants ranged in age from 40 to 50 years, and gender distribution was balanced (3 men and 2 women). 

Following the ISO 8587 (2007) [29] standard, the assessment criteria were based on sensory characteristics such as smell, vision, taste, and touch. The assessment comprehensively considered the color change, firmness, texture, and smell of the fruit during ripening. Mangoes transition from a green, hard state with a weak aroma to a yellow, soft, and juicy one with a strong aroma and eventually become dark with spots or mold. Peaches shift from green with a mild aroma to pink, soft, and juicy with a sweeter aroma and end up dark, possibly with mold or bruises. Bananas change from green with a subdued scent to yellow, soft, and creamy with a distinct aroma and later turn brown with potential black spots.

A scale of 1–10 was used for scoring, with higher scores indicating higher fruit ripeness. A score below 4 indicates that the fruit is unripe, a score of 4 to 7 indicates that the fruit is in the ripening stage, while a score above 7 means that the fruit is overripe. All scoring was performed in a sensory laboratory equipped with separate compartments according to ISO 8589 (2007) [30], and it was ensured that the evaluation process was carried out under standardized white light conditions to minimize interference from environmental factors.

### 2.5. GC-MS Analysis of the Characteristic VOCs Emitted by the Fruits during Different Ripening Stages

The characteristic VOCs were extracted using a solid-phase microextraction (SPME) method [31,32,33,34]. For each sample, 5 g of fruit was ground and added to a headspace vial. The non-polar divinylbenzene/carboxen/polydimethylsiloxane (DVB/CAR/PDMS) fibers then penetrated the septa to extract the volatiles for 40 min at 45 °C. GC-MS analysis was carried out on a 7890B GC System with a 7000C GC-MS Triple Quad mass detector (Agilent Technologies, Santa Clara, USA). The SPME fiber was quickly inserted into the GC-MS system and thermally desorbed at 250 °C for 3 min. Helium of high purity (99.999%) was used as the carrier gas, and the flow rate was maintained at 1.0 mL min^–1^. The column temperature was increased following a heating program, with an initial temperature of 50 °C maintained for 3 min, followed by a 10 °C min^–1^ ramp to 250 °C and a 5 min holding period. The mass spectra were analyzed using the NIST17.L library.

### 2.6. Preparation of the Characteristic VOCs

Gas mixtures were prepared according to previous methods [35], with the setup illustrated in Figure S2. Three mass flow controllers (CSC200-C, Sevenstar, Beijing, China) were employed to control the flow rate of mixture gas, including VOCs, dry N_2_, and wet N_2_, achieving different concentrations of the characteristic volatiles (Figure S2). The VOCs stream was maintained for 30 min in the pipelines to stabilize the analyte concentration. Subsequently, the colorimetric sensor arrays were placed within a gas chamber under exposure to the VOCs vapor.

### 2.7. Raw Data Acquisition and Process for the Characteristic VOCs

The sensor arrays were placed in the reaction chamber and reacted to the characteristic volatiles for 30 min, and the images of the sensor arrays before and after exposure were captured by a standard flatbed scanner (Epson Perfection V600, Seiko Epson Corporation, Suwa, Nagano, Japan). The RGB differential values (ΔR, ΔG, ΔB) of each spot in pre-exposure and post-exposure images were extracted and calculated using Python. The color differential maps were then generated by expanding the RGB range of 3–10 to 0–255. Euclidean Distance (ED = Euclidean Distance) was utilized as a quantitative measure to describe the detection response, obtained by subtracting the ΔR, ΔG, and ΔB values (ED = $\sqrt{{∆R}^{2}+∆G^{2}+∆B^{2}}$) [36,37,38]. HCA based on ED coupled with Ward’s minimum variance method was processed using the Origin software (OriginPro 2021, OriginLab Corporation, Northampton, MA, USA). All experimental data are the average of a minimum of three replicates.

### 2.8. Images Collection for Real Samples

In this study, each unripe fruit was placed in a transparent box with colorimetric sensor arrays attached to the interior surface of a transparent container (Figure S3). They were placed in a climatic chamber at a constant temperature of 25 °C and a humidity level of 95% which are common fruit storage conditions. Every 12 h, one image of each colorimetric sensor array was taken with a camera under arbitrarily transformed light and angles which can enhance the robustness of deep learning models by providing a comprehensive dataset. Finally, a dataset of 14,778 colorimetric sensor array images, to be used as a train and validation set, was collected to classify ripeness into ten classes: mango_unripe, mango_ripe, mango_overripe, peach_unripe, peach_ripe, peach_overripe, banana_unripe, banana_ripe, banana_overripe, and blank. The train and validation set was randomly selected from the dataset in a ratio of 17:3.

Separate test data, derived from different batches of fruits, was used to verify the robustness of the model. One image of each colorimetric sensor array was captured using a camera under arbitrarily transformed light and angles every 12 h. Finally, a dataset of 1370 colorimetric sensor images documenting the progression of fruits from their unripe to overripe stages was collected as the test set.

### 2.9. DenseNet Model Architecture

The DenseNet model (Figure S4) consists of an input layer, multiple convolutional layers, a spatial pyramid pooling (SPP) structure, a fully connected layer, and an output layer. This model utilizes five identical dense blocks, each of which contain three 3 × 3 convolutional layers, batch normalization, and a ReLU activation function, with a growth rate of 32. A transition module is used between every two dense blocks for downsampling transformation, consisting of a batch normalization layer, 1 × 1 convolutional layer, and 2 × 2 avgpooling.

### 2.10. Data Process for Deep Learning

For image processing, all the images were resized to 255 × 255 pixels as input. Four DCNN models, google inception net (GoogleNet) [39], inception version 3 (Inception_v3) [40], residual network 18 (ResNet18) [41], and DenseNet models were implemented in Pytorch (1.8.1 + cu111). All model hyperparameters were set equally. The models implemented cross-entropy loss as the loss function, stochastic gradient descent (SGD) as the optimizer, momentum as 0.9, and weight decay as 0.0001. The training process consisted of 500 epochs with a learning rate scheduler for stable performance. The learning rate was initially set to 0.01 and was gradually reduced to 0.005 between the 300th and 450th epochs, and then to 0.001 for the remaining epochs. A batch size of 256 was used for the train set. DCNN models were implemented using Python on a computer equipped with RTX 3090 GPUs.

## 3. Results and Discussion

### 3.1. GC-MS Analysis of Fruit Characteristic Volatiles at Different Ripening Stages

The degree of banana ripeness was determined by the integration of firmness test and sensory evaluation. It was observed that banana firmness (Figure S5c) decreased progressively with increased storage time, with the rate showing significant changes at 2 days and 6 days. The color transformation of bananas was documented (Figure S6), transitioning from light green (unripe) to yellow (ripe), and ultimately to brown (overripe). Average sensory evaluations indicated that bananas were generally unripe between 0 ≤ days < 2, ripe between 2 ≤ days < 6, and overripe between 6 ≤ days ≤ 8 according to sensory score ratings, as detailed in Table S2. These sensory stages correspond closely with the observed changes in banana firmness. Likewise, mangoes were classified into three ripeness stages based on the firmness test (Figure S5a), and sensory evaluation (Table S3, Figure S7), with 0 ≤ days < 3 for unripe, 3 ≤ days < 8 for ripe, and 8 ≤ days ≤ 10 for overripe. Peaches were classified into three ripeness stages based on the firmness test (Figure S5b), and sensory evaluation (Table S4, Figure S8), with 0 ≤ days < 2 for unripe, 2 ≤ days < 7 for ripe, and 7 ≤ days ≤ 10 for overripe.

The VOCs released from the fruit (mango, peach, and banana) during different ripening stages were monitored using the GC-MS method. According to the relative abundances and variation patterns at various ripening stages, the representative characteristic VOCs were identified (Table S5) [42,43,44,45,46,47,48,49]. (+)-limonene, β-myrcene, and 3-carene were identified as the characteristic VOCs for mango. Benzaldehyde, ethyl acetate, and hexyl acetate were identified as the characteristic VOCs for peach. Ethanol, trans-2-hexenal, and isoamyl acetate were identified as the characteristic VOCs for banana.

### 3.2. Sensor Response to the Individual Gas Analyte

The gas-sensitive dyes for fabricating the colorimetric sensor arrays were selected according to the characteristic VOCs released from the fruit. The identified characteristic VOCs are classified as hydrocarbons, esters, alcohols, and aldehydes. Thus, a diverse range of dye types was selected, including two aldehyde/ketone-sensitive dyes, three solvatochromic dyes, three redox dyes, three Lewis acidic dyes, and fourteen pH indicators, which are sensitive to the aforementioned characteristic VOCs.

**Figure 2:**
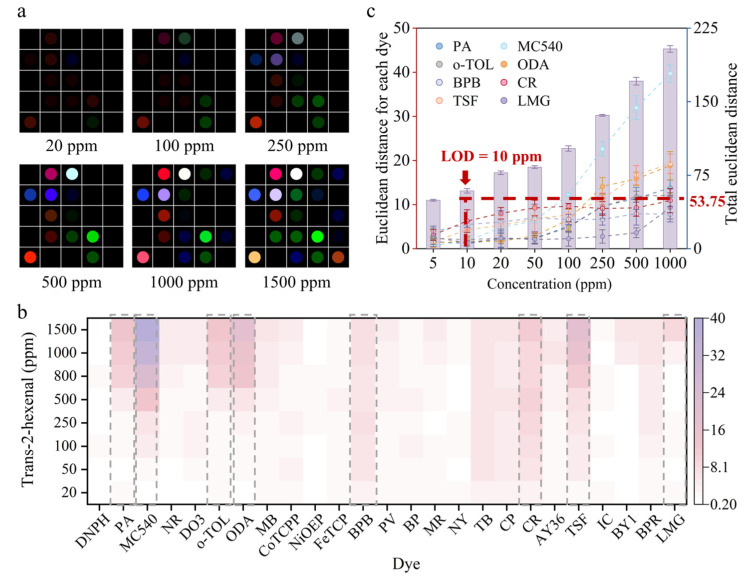
Colorimetric sensor array response to trans-2-hexenal. (**a**) Color differential maps were obtained by extracting R, G, and B values from the images at concentrations of 20 ppm, 100 ppm, 250 ppm, 500 ppm, 1000 ppm, and 1500 ppm of trans-2-hexenal, the RGB range is expanded from 3–10 to 0–255. (**b**) ED heatmaps of different concentrations of trans-2-hexenal. (**c**) The ED values of sensitive dyes in response to trans-2-hexenal, similar to the total ED of the colorimetric sensor array, show an increase with increasing concentrations of trans-2-hexenal.

The fabricated colorimetric sensor arrays were employed to detect the various concentrations of fruit characteristic VOCs. Under exposure to trans-2-hexenal with concentrations ranging from 3–1000 ppm (Figure S9), the images of the colorimetric sensor arrays exhibited color change. The RGB differential maps of trans-2-hexenal at concentrations of 20 ppm, 100 ppm, 250 ppm, 500 ppm, 1000 ppm, and 1500 ppm were obtained by subtracting the pre-exposure image from each post-exposure image (Figure 2a). As the trans-2-hexenal concentrations increased, the RGB differential map showed deeper color, implying concentration-dependent response characteristics of the colorimetric sensor arrays.

An ED heatmap analysis was performed to show the intensity variation (Figure 2b). ED was utilized as a quantitative measure to describe the signal intensity obtained by extracting the ΔR, ΔG, and ΔB values (ED = $\sqrt{{∆R}^{2}+∆G^{2}+∆B^{2}}$). Based on the heatmap analysis, dyes of pararosaniline, merocyanine 540, o-tolidine, o-dianisidine, bromophenol blue, cresol red, 3,3,5,5-tetraiodophenolsulfonphthalein, and leuco malachite green were identified as the most sensitive dyes to trans-2-hexenal. The ED values of these sensitive dyes to trans-2-hexenal followed a similar trend to the total ED values of the colorimetric sensing combinatorics, both exhibiting a noticeable increase with the concentration of trans-2-hexenal (Figure 2c). The limit of detection (LOD) was the minimum concentration whose corresponding ED value is above the mean of the blank control (ED_blank_ = 47.75) plus three times its standard deviation (3σ = 6). The LOD for trans-2-hexenal was found to be 10 ppm (Figure 2c).

### 3.3. Classification Performance for the Multiple VOCs 

To evaluate the capacity of the sensor arrays to distinguish multiple VOCs, another eight characteristic VOCs including isoamyl acetate, benzaldehyde, hexyl acetate, ethanol, ethyl acetate, 3-carene, β-myrcene, and (+)-limonene were also tested. As shown in ED heatmaps, the ED value increased with the enhanced VOC concentrations and all of the VOCs showed different intensity variations (Figure S10). 

**Figure 3:**
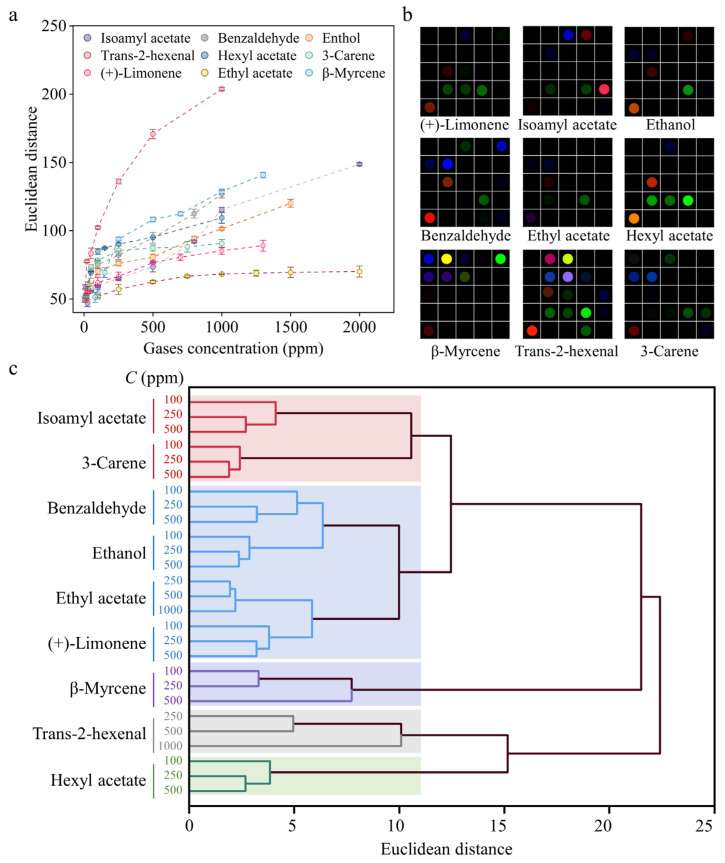
Feasibility of using the colorimetric sensing combinatorics to detect multiple VOCs emitted by fruit. (**a**) ED value of the colorimetric sensing combinatorics increases with the rising concentration of the characteristic gases. (**b**) Color differential maps were obtained by extracting R, G, and B values from the images at concentrations of 500 ppm of nine characteristic gases, the RGB range is expanded from 3–10 to 0–255. (**c**) HCA produced a cluster tree that showed the nine gases could be distinguished at different concentrations, with a 100% success rate.

As the concentration of the characteristic VOCs increases, the total ED value of the colorimetric sensing combinatorics rises, with the LOD established using ED for isoamyl acetate (50 ppm), benzaldehyde (20 ppm), hexyl acetate (25 ppm), ethanol (50 ppm), ethyl acetate (250 ppm), 3-carene (25 ppm), β-myrcene (100 ppm), and (+)-limonene (25 ppm) (Figure 3a). Distinguishable patterns for all nine characteristic VOCs at 500 ppm were observed (Figure 3b), demonstrating the capacity of the colorimetric sensor arrays to distinguish between multiple VOCs. 

HCA, as a statistical method for grouping data points into clusters based on their similarity, was used to evaluate the distinguishing performance of the colorimetric sensor arrays for the nine characteristic VOCs with different concentrations. Ward’s minimum variance method was used to determine gas-induced variations of the 27 × 25 (9 VOCs × 3 concentrations × 25 ED values) dimensional matrix. The resulting cluster-tree showed that the nine VOCs with different concentrations formed clearly separate clusters with a 100% success rate (Figure 3c). Together, these findings suggest that it is possible to use colorimetric sensor arrays to monitor fruit ripeness.

### 3.4. Deep Learning-Enabled Fruit Ripeness Recognition

As a proof of concept, the colorimetric sensor arrays were employed to monitor the fruit ripeness of real samples. The colorimetric sensor arrays were attached to a transparent container containing fruit (Figure S3), maintained at a steady temperature of 25 °C and a humidity level of 95%. Images of the sensor arrays were captured at 12 h intervals using a camera. However, in a real fruit ripening detection environment, variations in light and shooting angles present challenges for accurately classifying fruit ripeness levels. 

The traditional classified method (ED) cannot solve the problem. To establish a reference range, this work measured the firmness values and sensory evaluations, and calculated the ED values of ten samples for each fruit variety stored at different ripeness levels [25]. Before calculating the ED values, all images were calibrated for color balancing to ensure accurate color extraction under arbitrary lighting conditions according to the marked white and black labels [50] using Photoshop software (Adobe Photoshop Version 22.4.3, Adobe Inc., San Jose, CA, USA). A comparative analysis of pre- and post-image calibration is presented in Figure S11. After color calibration, the dye’s RGB values exhibit closer similarity under varied lighting conditions, showing the effectiveness of color calibration. Based on sensory evaluation and firmness data, fruits were categorized into ten distinct classes for classification tasks: mango_unripe, mango_ripe, mango_overripe, peach_unripe, peach_ripe, peach_overripe, banana_unripe, banana_ripe, banana_overripe, and blank. The ED values’ reference range (Table S6) for each category of fruit ripeness was established accordingly, which was utilized to forecast the ripeness of unknown fruit samples. For each category, twenty colorimetric sensor images were randomly selected to evaluate the prediction accuracy of the ED method based on the ED values’ reference range. However, ED analysis showed an overall accuracy rate of 74.50% (Figure S12), indicating great challenges in distinguishing fruit ripeness. The low accuracy can be attributed to the fact that the values of ED can be affected by photographic conditions such as lighting, zoom, and angle.

**Figure 4:**
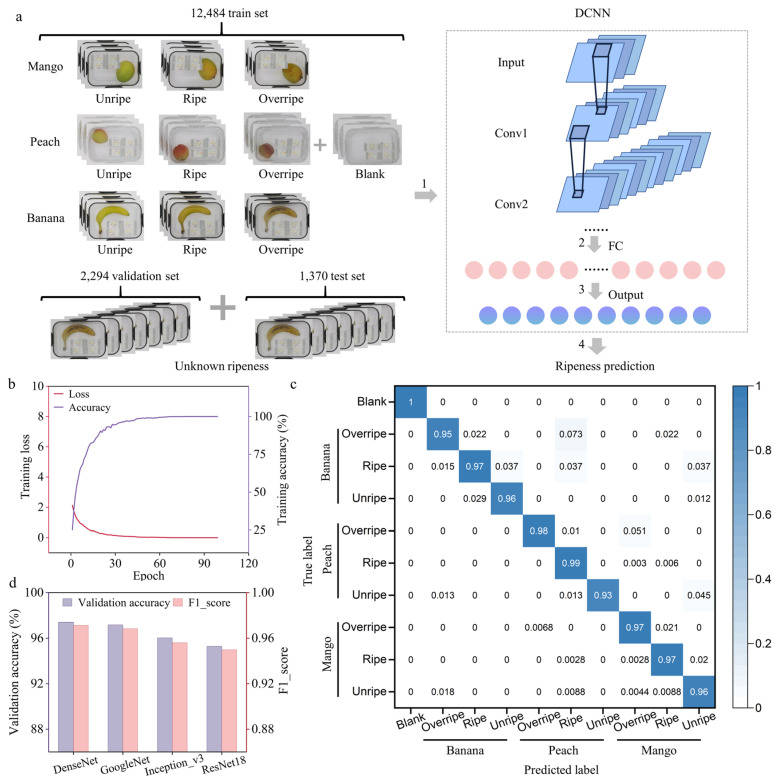
Colorimetric sensor detection combined with DCNN to detect fruit ripeness. (**a**) Schematic diagram of fruit ripeness label classification and deep learning for fruit ripeness recognition. (**b**) The training loss and the training accuracy of the DenseNet model. The training loss measures the dissimilarity between the predicted and actual results. The training accuracy increases with the number of epochs. (**c**) Confusion matrix of DenseNet for the validation set. (**d**) Comparison of the validation accuracy and F1_score on the validation set between four DCNN models (DenseNet, GoogleNet, Inception_v3, and ResNet18).

Therefore, DCNN models, as a powerful method, were used to identify multidimensional image data and reduce the impact of varied environmental conditions. The dataset consists of 12,484 images for training, 2294 images for validation, and 1370 images for testing (Figure 4a). A ten-category image classification network was designed with various DCNN backbones, each consisting of an input layer, multiple convolution (conv.) layers, fully connected layers (FC), and an output layer. The trained DCNNs efficiently extracted features from colorimetric sensor images. After training, the accuracy of the models’ classification of the array images was evaluated using the validation and test set. When an image was input into the classification network, the system provided the most likely ripeness category for the fruit (blue circles in Figure 4a). 

Four models, including DenseNet, GoogleNet, Inception_v3, and ResNet18, were implemented. The GoogleNet model uses “Inception” modules for efficient image feature processing. Inception_v3, an evolution of GoogleNet, utilizes more layers and diverse kernel sizes to enhance feature extraction and efficiency. ResNet18 utilizes residual blocks with cross-layer connections to improve information flow. DenseNet, detailed in our methodology, utilizes dense connections to promote feature reuse and address the vanishing-gradient problem, thereby improving efficiency and performance significantly. With all model hyperparameters set equally, the performance of the four models is compared in terms of training and validation accuracy, F1_score, and test accuracy (Table 1).

The accuracy of the training and validation set was first compared. Examination of the accuracy-loss graphs revealed that, with an increasing number of training epochs, training accuracy improved, and training loss decreased, approaching zero as illustrated in (Figure 4b and Figure S13). All models converged within 200 epochs, achieving peak ripeness prediction accuracy, and indicating effective training. The confusion matrices for the four DCNN models were compared, whose diagonals show the correctly classified sample ratios (Figure 4c and Figures S14–S16). The DenseNet model outperformed others on the validation set with a 97.39% accuracy, followed by GoogleNet, Inception_v3, and ResNet18 with 97.17%, 96.03%, and 95.29% (Figure 4d and Table 1), respectively. 

F1_score as an indicator of accuracy was also used to evaluate and compare the performance of the DCNN models, using the following equation:F1_score=2pr/(p+r), where “p” and “r” denote precision and recall, respectively. F1_score is an ideal comprehensive metric that considers both precision and recall simultaneously, making it a better choice when seeking a balance between precision and recall. The DenseNet model exhibited the highest F1_score with 0.9712 among the compared models followed by GoogleNet, Inception_v3, and ResNet18 with 0.9683, 0.9560, and 0.9498, respectively, (Figure 4d and Table 1). 

The accuracy of the separate test set for the four models was then compared. Test data derived from different batches of fruit evaluates the robustness of the model. The DenseNet model outperformed others on the test set in predicting fruit ripeness with an 82.20% accuracy, followed by GoogleNet, Inception_v3, and ResNet18 with 78.85%, 78.63%, and 76.73% (Table 1), respectively, demonstrating the superior robustness of the DenseNet model. Compared to previous studies, the test accuracy in our work is better or comparable with that of the previous literature (Table S7) [51,52,53,54,55,56].

Due to the DenseNet model’s outstanding performance, Gradient-weighted Class Activation Mapping (Grad-CAM) was applied to provide a more intuitive understanding of this model’s decision-making process. The visual class heatmap, acquired based on the attention mechanism [57], can indicate the features that have a more important impact on the results through darker colors. Heatmaps of the ripeness stages of mangoes (unripe, ripe, and overripe) were analyzed and obtained using Grad-CAM (Figure S17). These maps showed the prominent features affecting the model. The dyes including nile red, disperse orange #3, o-tolidine, m-cresol purple, indigo carmine, basic yellow1, and leuco malachite green are the most influential and sensitive dyes in classifying mango ripeness, and they contribute the most to the final decision. The above results demonstrate the interpretability of the DenseNet model.

All of these results show that DenseNet has superior performance when classifying the olfactory visualized ripeness of fruit. This exceptional ability is mainly attributed to the efficient use of the dense connections in the DenseNet model. These connections not only improve feature extraction and reuse but also significantly reduce the risk of overfitting, which is effective in addressing complex classification tasks.

Overall, our system combines colorimetric sensing arrays with the DenseNet model to successfully identify fruit ripeness with a validation set accuracy of 97.39%. This approach offers numerous advantages when compared to other well-known methods. As seen in Table 2, our method not only classified the ripeness of various kinds of fruits but also had higher accuracy than other well-known methods.

## 4. Conclusions

In summary, this study presents a new method for the non-destructive monitoring of fruit ripeness by integrating cross-reactive colorimetric sensing combinatorics with the DenseNet model. The colorimetric sensing combinatorics consisted of 25 dyes that were sensitive to volatile gases emitted by fruits, showing cross-reactivity to various types and concentrations of gases. The formation of a unique scent fingerprint can be identified using DCNN. By training on 12,484 images under varying lighting conditions, the DenseNet could learn autonomously and mitigate the impact of illumination on the experimental results, achieving an impressive accuracy rate of 97.39% on the validation test and 82.20% on the test set.

With its attractive features of portability, low cost, and high accuracy, this system has great potential for integration into existing smart packaging or monitoring systems. It provides a simple and non-destructive pfigureattern for ordinary consumers to evaluate the fruit ripeness and benefits the supply chain, especially for costly fruits from farmers and producers. However, there are still challenges for its commercial application. Current testing devices are not portable enough for on-site testing. Furthermore, various fruit varieties emit different complex VOCs. For a new fruit variety, a large number of data samples are required for training using deep learning models to achieve a commercial application. 

Looking forward, we anticipate significant advancements in sensor technologies and analytical methods to mitigate these challenges. Enhanced sensor sensitivity and anti-interference capability, coupled with more advanced data analysis techniques, could significantly improve the system’s adaptability, and reduce the need for large datasets. In perspective, such technological and collaborative advancements not only promise to refine the system’s current capabilities but also to broaden its utility across various fields, including food safety and quality assurance.

## Figures and Tables

**Table 1:** Comparison results among the four DCNN models.

Models	Validation Accuracy (%)	F1_Score	Test Accuracy (%)
DenseNet	97.39	0.9712	82.20
GoogleNet	97.17	0.9683	78.85
Inception_v3	96.03	0.9560	78.63
ResNet18	95.29	0.9498	76.73

**Table 2:** Comparison with other well-known methods of fruit ripeness classification.

Method	Fruit	Classification Method	Accuracy/Correlation Coefficient	Ref.
RGB	Mango	Fuzzy logic	87%	[58]
VIS/NIR	Watermelon	ANN	80%	[59]
Acoustic method	Mango	-	0.957	[60]
Electronic Nose	Peaches and pears	-	92%	[61]
RGB	Blueberry	KNN, etc.	85–98%	[62]
HSV	Mango	Neural Network	95%	[63]
Electrical method	Banana	-	0.94	[64]
RGB	Mango	Gaussian Mixture model and Fuzzy logic	Less than 90% in all varieties	[65]
Bioimpedance data	Strawberry	MLP network	0.72	[66]
Colorimetric sensor arrays	Banana, Peach, Mango	DenseNet	97.39%	This study

## Data Availability

The data presented in this study are available on request from the corresponding author. The data are not publicly available due to institutional policy regarding data protection.

## Supplementary Materials

The following supporting information can be downloaded at: https://www.mdpi.com/article/10.3390/foods13050793/s1, Figure S1. Working mechanism of the (a) human olfactory and (b) olfactory visualization system utilizing color sensing combinateries with DCNN. Figure S2. Gas distribution device. Figure S3. (a) Schematic diagram and (b) photograph of the fruit packaged transparent container with colorimetric sensor arrays. Figure S4. Overview of DenseNet model architecture. Figure S5. Firmness variation of (a) mango, (b) peach, and (c) banana during the storage time. Figure S6. Appearance of banana changes with the storage time. Figure S7. Appearance of mango changes with the storage time. Figure S8. Appearance of peach changes with the storage time. Figure S9. Images of the colorimetric sensor array’s response to the trans-2-hexenal with different concentrations range from 3 to 1000 ppm. Figure S10. ED heatmaps of the characteristic VOCs with different concentrations. Figure S11. (a–c) Color balancing performed by internal calibration makers under various light conditions. Changes in the RGB values of pararosaniline dye as represented by images (d) before color calibration and (e) after color calibration. Figure S12. Detection accuracy rate for fruit ripeness based on ED calculations after color calibration. Figure S13. Training loss and training accuracy of the (a) GoogleNet, (b) Inception_v3, and (c) ResNet18 model. Figure S14. Confusion matrix of GoogleNet for validation set. Figure S15. Confusion matrix of Inception_v3 for validation set. Figure S16. Confusion matrix of ResNet18 for validation set. Figure S17. Attention mechanism maps of (a) unripe, (b) ripe, and (c) overripe mangoes. Table S1: Types, serial numbers, corresponding names, and abbreviations of dyes in literature. Table S2. Average sensory score of the banana during storage for each group. Table S3. Average sensory score of the mango during storage for each group. Table S4. Average sensory score of the peach during storage for each group. Table S5. Identification of VOCs and their relative abundances released from fruit during storage time by GC–MS. Table S6. ED values’ reference range to three ripeness levels. Table S7. Comparsion of test accuracy among this study with previous studies.
