# Supplementary material for: Integrated Fruit Ripeness Assessment System Based on an Artificial Olfactory Sensor and Deep Learning
Source: Foods. 2024 Mar 4;13(5):793. doi: 10.3390/foods13050793 (PMC10931156; doi:10.3390/foods13050793)
Supplement: Supplementary file 1 [file foods-13-00793-s001.zip › foods-2869952-supplementary.pdf]

Supporting information

Table S1. Types, serial numbers, corresponding names, and abbreviations of dyes in literature.

Type	Number	Name	Abbreviation
Aldehyde/ketone-sensitive dyes	1	2,4-Dinitrophenylhydrazine	DNPH
	2	Pararosaniline	PA
Solvatochromic dyes	3	Merocyanine 540	MC540
	4	Nile red	NR
	5	Disperse orange #3	DO3
Redox dyes	6	o-Tolidine	o-TOL
	7	o-Dianisidine	ODA
	8	Methylene blue	MB
Lewis acidic dyes	9	CoTCPP	--
	10	2,3,7,8,12,13,17,18-Octaethyl-21H,23H porphine nickel(II)	NiOEP
	11	FeTcp	--
pH indicators	12	Bromophenol blue	BPB
	13	Pyrocatechol violet	PV
	14	Bromocresol purple	BP
	15	Methyl red	MR
	16	Nitrazine yellow	NY
	17	Thymol blue	TB
	18	m-Cresol Purple	CP
	19	Cresol red	CR
	20	Acid yellow 36	AY 36
	21	3,3,5,5-Tetraiodophenolsulfonphthalein	TSF
	22	Indigo carmine	IC
	23	Basic yellow1	BY1
	24	Bromopyrogallol red	BPR
	25	Leuco malachite green	LMG

Table S2. Average sensory score of the banana during storage for each group. Evaluator A-E represent our study's participants, who were aged between 40 to 50 years and included three men and two women.

Storage time (D)	Evaluator A	Evaluator B	Evaluator C	Evaluator D	Evaluator E	Average value
0	1	2	0	1	2	1.2
1	3	2	1	1	3	2
2	5	5	3	4	4	4.2
3	6	5	4	6	5	5.2
4	7	6	5	5	6	5.8
5	6	7	5	6	7	6.2
6	7	8	8	7	8	7.6
7	9	9	10	9	9	9.2
8	10	9	10	10	10	9.8

0~3: unripe; 4~7: ripe; 8-10: overripe.

Table S3. Average sensory score of the mango during storage for each group. Evaluator A-E represent our study's participants, who were aged between 40 to 50 years and included three men and two women.

Storage time (D)	Evaluator A	Evaluator B	Evaluator C	Evaluator D	Evaluator E	Average value
0	1	0	1	2	0	0.8
1	1	2	0	1	2	1.2
2	2	2	3	1	3	2.2
3	4	5	4	4	3	4.0
4	4	5	3	5	4	4.2
5	6	4	5	4	5	4.8
6	6	7	4	5	7	5.8
7	6	7	6	5	7	6.2
8	7	8	8	7	8	7.6
9	8	10	8	9	9	8.8
10	9	10	9	10	10	9.6

0~3: unripe; 4~7: ripe; 8-10: overripe.

Table S4. Average sensory score of the peach during storage for each group. Evaluator A-E represent our study's participants, who were aged between 40 to 50 years and included three men and two women.

Storage time (D)	Evaluator A	Evaluator B	Evaluator C	Evaluator D	Evaluator E	Average value
0	1	2	0	1	0	0.8
1	2	3	1	2	3	2.2
2	4	4	3	4	5	4
3	4	5	4	5	5	4.6
4	5	5	5	6	5	5.2
5	6	6	5	4	6	5.4
6	6	7	5	5	6	5.8
7	8	8	6	7	7	7.2
8	7	8	7	9	8	7.8
9	8	8	9	10	8	8.6
10	9	9	10	10	9	9.4

0~3: unripe; 4~7: ripe; 8-10: overripe.

Table S5. Identification of VOCs and their relative abundances released from fruit during storage time by GC–MS.

Fruit	Compound	Relative abundance (%)			Match factor	Ref.
		Unripe	Ripe	Overripe		
Mango	3-carene	-	47.36	33.22	97.9	[42]
	(+)-limonene	7.95	6.35	4.68	98.3	[43]
	β-myrcene	8.91	8.40	4.10	87.6	[44]
Peach	benzaldehyde	1.40	43.58	20.09	91.3	[45]
	ethyl acetate	-	-	33.67	90.0	[46]
	hexyl acetate	-	-	10.76	90.4	[47]
Banana	ethanol	11.06	1.50	3.16	74.0	[48]
	trans-2-hexenal	-	9.67	-	97.0	[49]
	isoamyl acetate	-	8.90	7.28	90.0	[49]

"-" indicates that it is not detected.

Table S6. ED values' reference range to three ripeness levels.

Ripeness	Mango	Peach	Banana
Unripe	<81.63	<72.12	<85.15
Ripe	81.63-136.15	72.12-126.31	85.15-144.32
Overripe	>136.15	>126.31	>144.32

The ED value of each sample was obtained after color calibration. This study firstly labels the ripeness of each fruit sample with different storage intervals based on firmness values and sensory evaluation. According to the ripeness of each sample, the ED value ranges corresponding to the ripeness level are obtained. These ED value ranges are utilized for the prediction of the ripeness state of other samples.

Table S7. Comparsion of test accuracy among this study with previous studies.

Method	Test accuracy (%)	Ref.
DenseNet	82.20	This study
Part-A^2-anchor	79.47	[51]
BEVFusion	75.0	[52]
PointRCNN	57.94	[53]
Part-A^2	61.79	[54]
PV-RCNN	62.81	[55]
F-PointNet	56.02	[56]

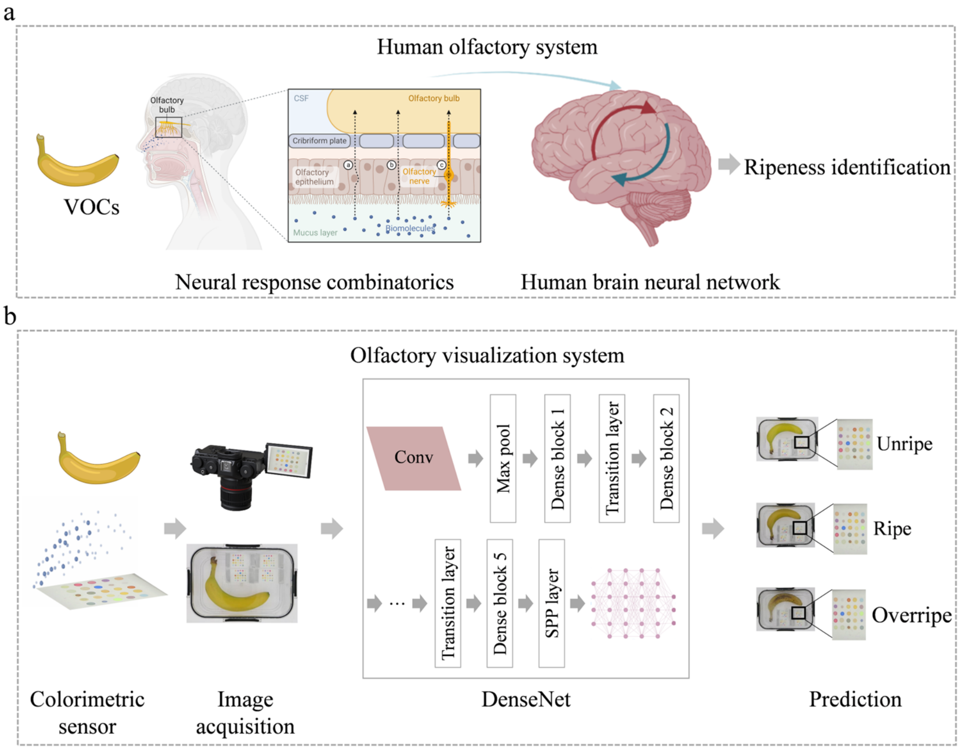

Figure S1. Working mechanism of the (a) human olfactory and (b) olfactory visualization system utilizing color sensing combinateries with DCNN.

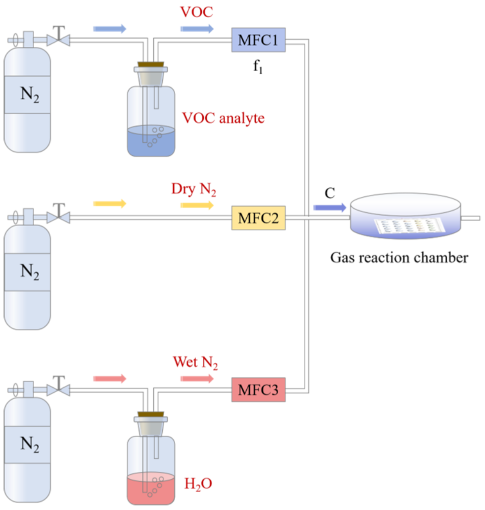

Figure S2. Gas distribution device. Different concentrations of a gas can be generated by mixing a known concentration of the gas with nitrogen gas. The concentration of the resulting diluted VOCs can be calculated using the following equation, where C represents the concentration of the diluted VOCs (ppm), C_1 represents the original concentration of the VOCs (ppm), f_1 represents the flow rate of the pipeline containing the VOCs (mL/min), and f_2 represents the flow rate of the nitrogen gas in the other pipelines (mL/min):

$$C = \frac{C_1 \times f_1}{f_1 + f_2}$$

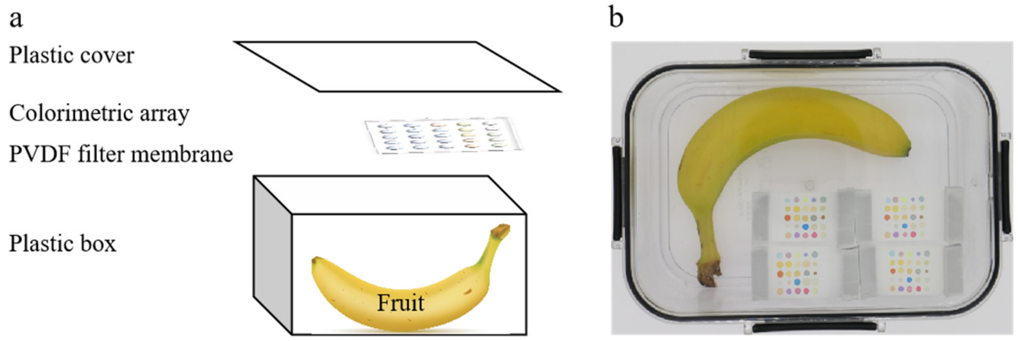

Figure S3. (a) Schematic diagram and (b) photograph of the fruit packaged transparent container with colorimetric sensor arrays.

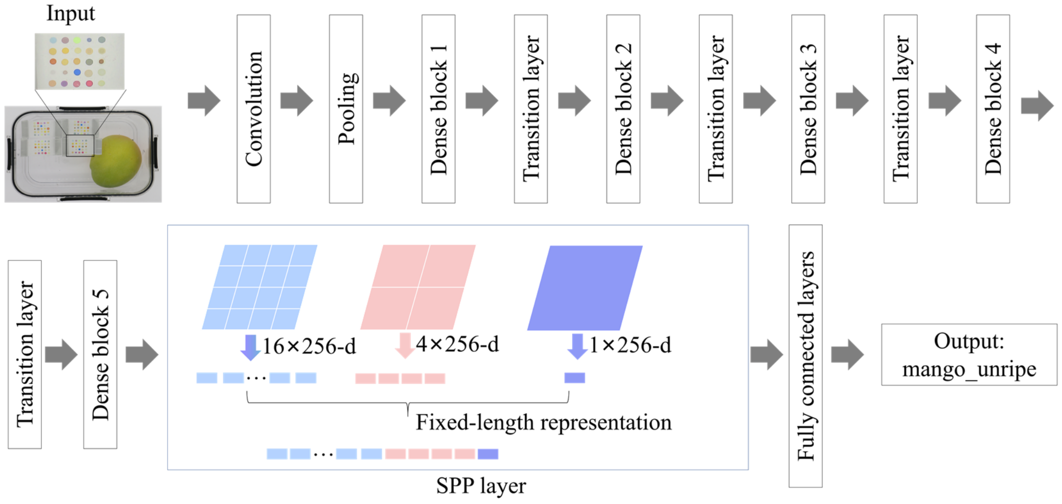

Figure S4. Overview of DenseNet model architecture. The DenseNet model framework is demonstrated using the input of the unripe mango label as an example.

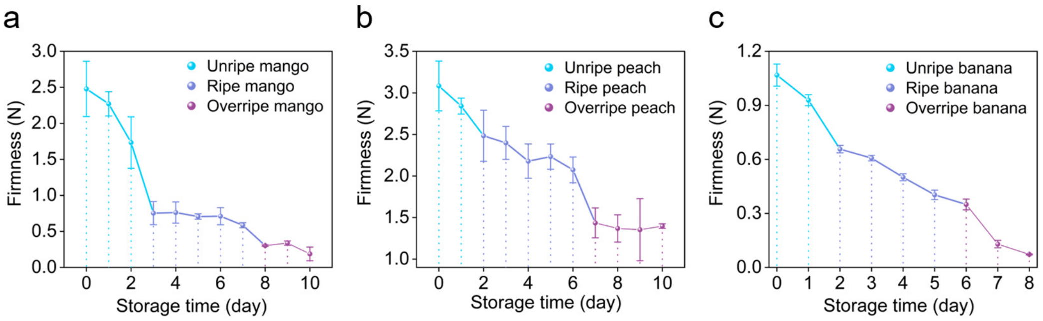

Figure S5. Firmness variation of (a) mango, (b) peach, and (c) banana during the storage time.

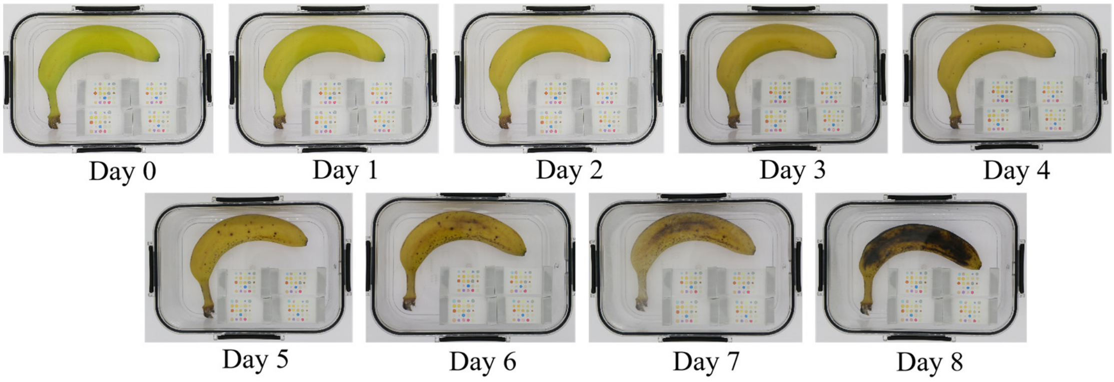

Figure S6. Appearance of banana changes with the storage time.

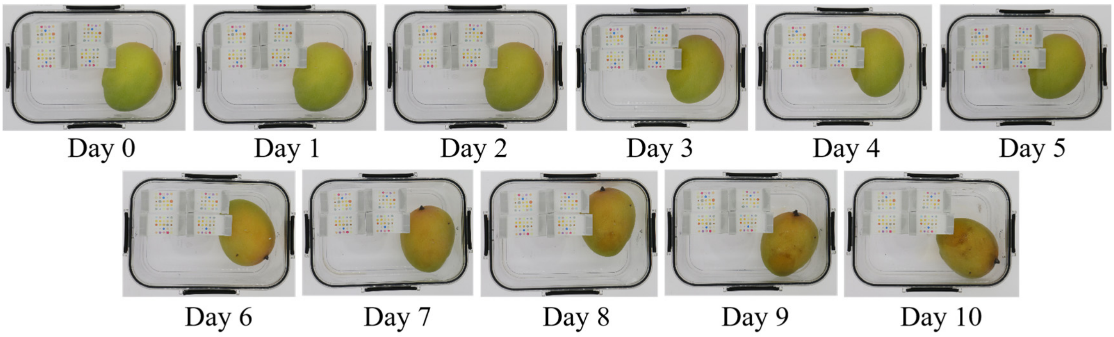

Figure S7. Appearance of mango changes with the storage time.

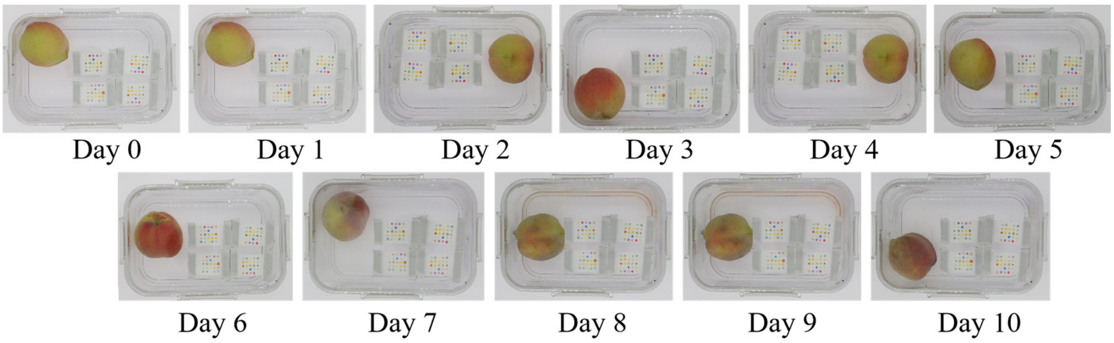

Figure S8. Appearance of peach changes with the storage time.

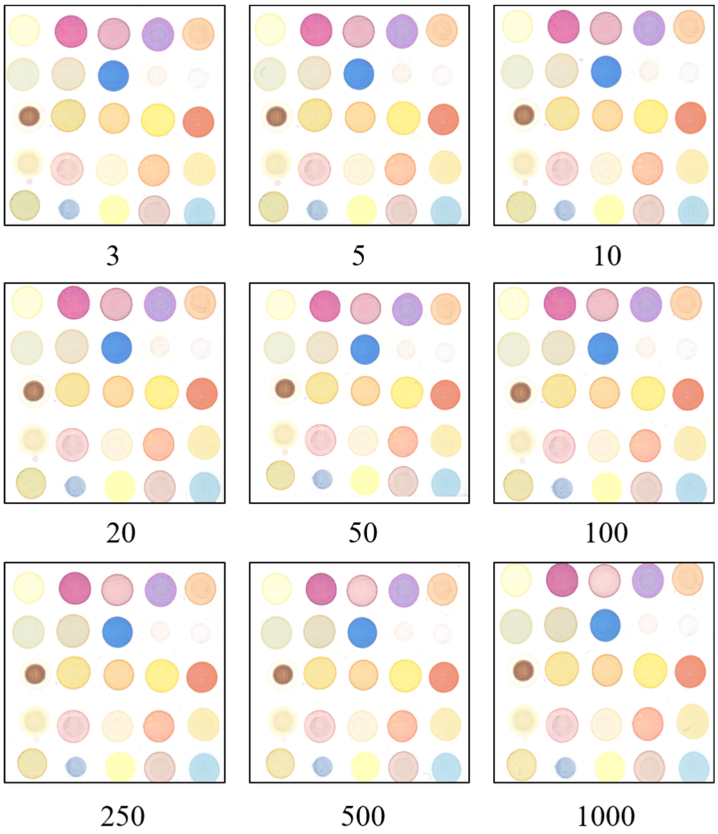

Figure S9. Images of the colorimetric sensor array's response to the trans-2-hexenal with different concentrations range from 3 to 1000 ppm.

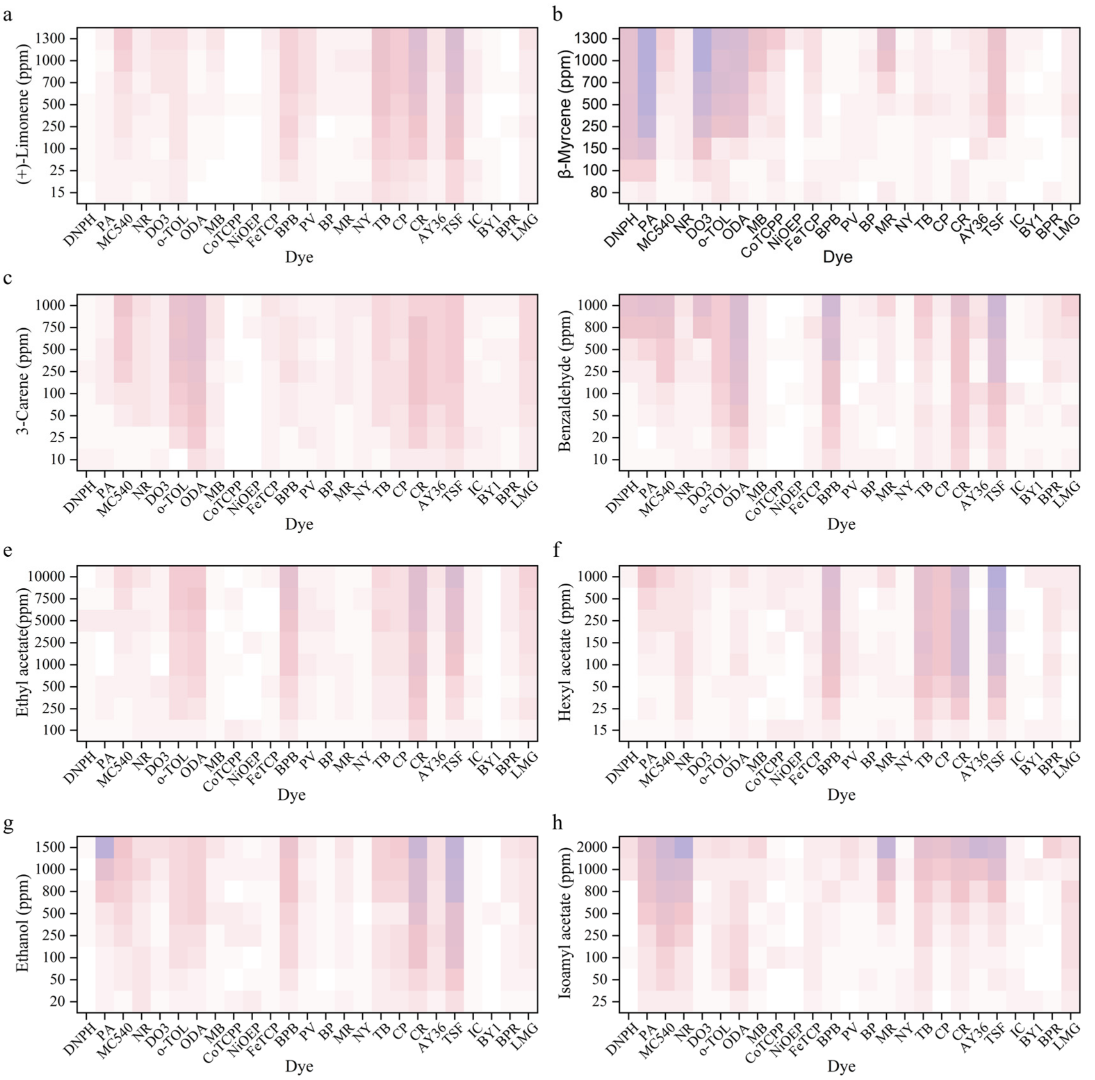

Figure S10. ED heatmaps of the characteristic VOCs with different concentrations, including (a) (+)-limonene, (b) β-myrcene, (c) 3-carene, (d) benzaldehyde, (e) ethyl acetate, (f) hexyl acetate, (g) ethanol, and (h) isoamyl acetate.

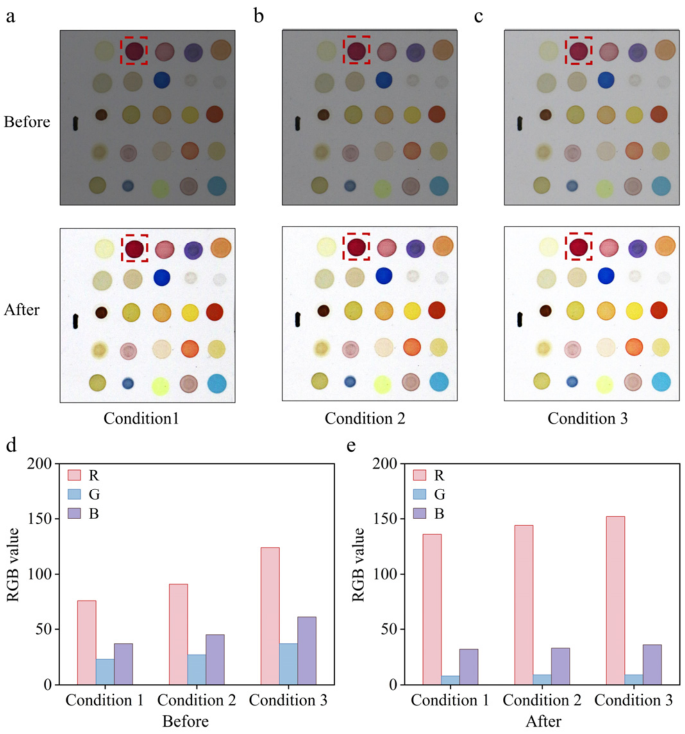

Figure S11. (a-c) Color balancing performed by internal calibration makers under various light conditions. Changes in the RGB values of pararosaniline dye as represented by images (d) before color calibration and (e) after color calibration.

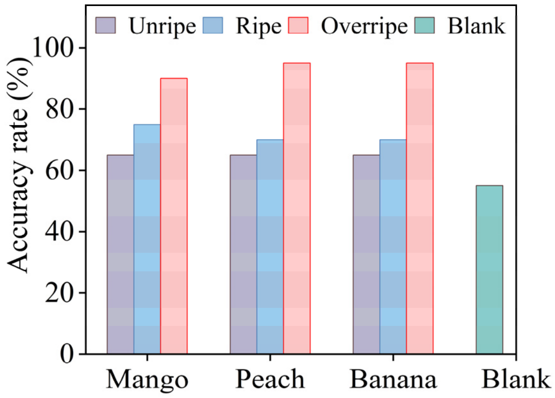

Figure S12. Detection accuracy rate for fruit ripeness based on ED calculations after color calibration.

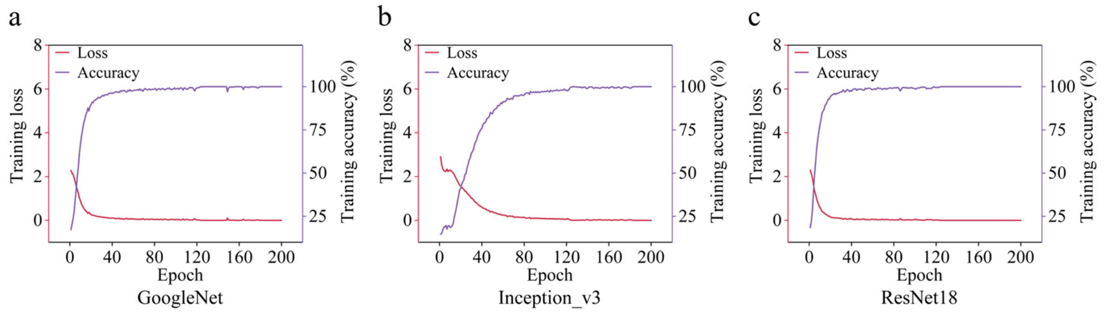

Figure S13. Training loss and training accuracy of the (a) GoogleNet, (b) Inception_v3, and (c) ResNet18 model.

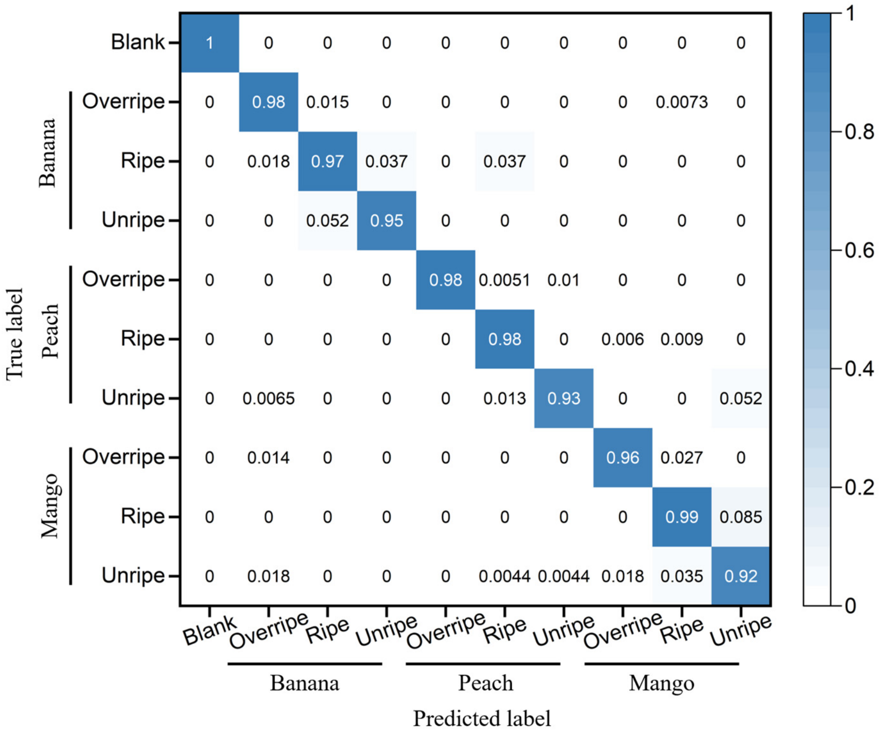

Figure S14. Confusion matrix of GoogleNet for validation set.

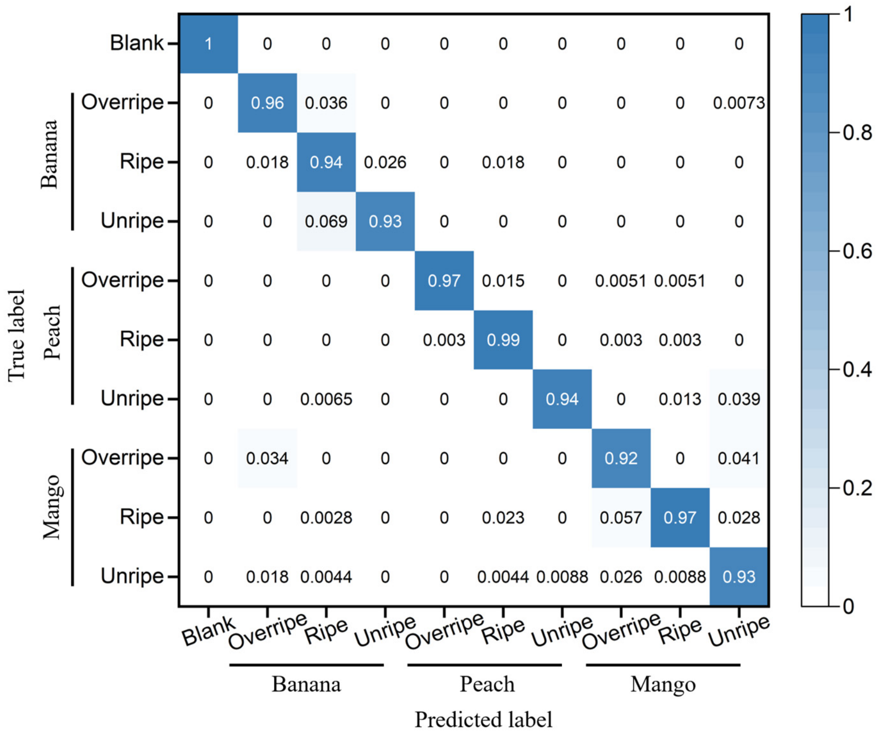

Figure S15. Confusion matrix of Inception_v3 for validation set.

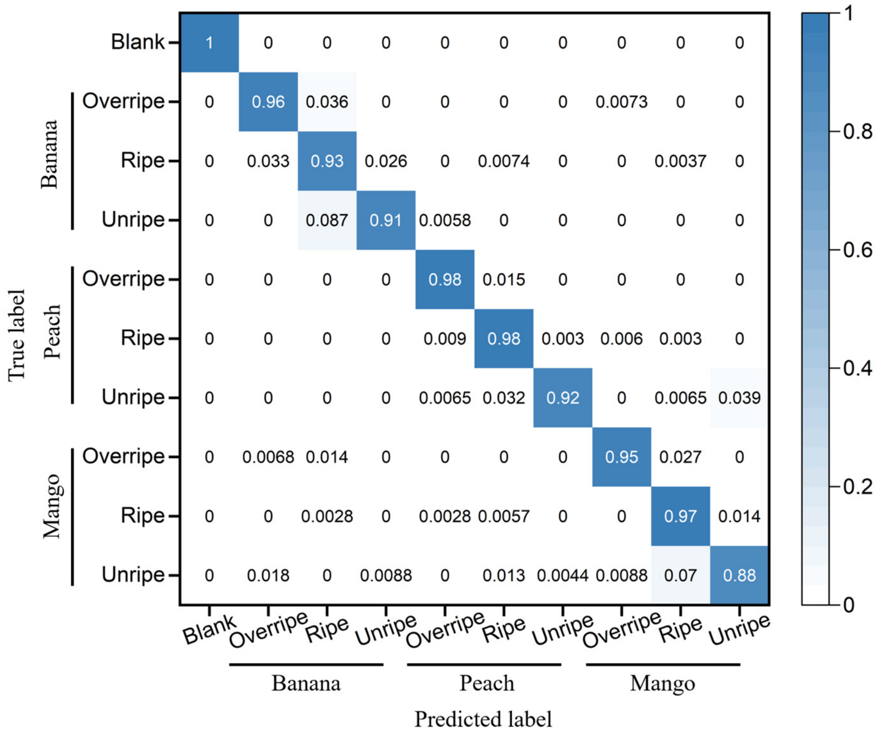

Figure S16. Confusion matrix of ResNet18 for validation set.

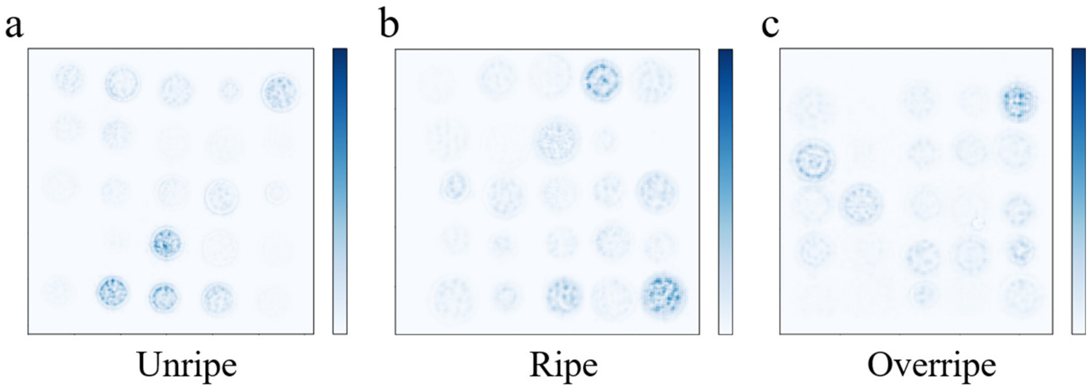

Figure S17. Attention mechanism maps of (a) unripe, (b) ripe, and (c) overripe mangoes. The
